# Flexible Temperature Sensors on Fibers

**DOI:** 10.3390/s100907934

**Published:** 2010-08-26

**Authors:** Maciej Sibinski, Malgorzata Jakubowska, Marcin Sloma

**Affiliations:** 1 Department of Semiconductor and Optoelectronics Devices, Technical University of Lodz/211/215 Wolczanska Street, 90-924 Lodz, Poland; 2 Faculty of Mechatronicsm, Warsaw University of Technology/Sw. Andrzeja Boboli str. 8, 02-525 Warsaw, Poland; E-Mail: marcin.sloma@mchtr.pw.edu.pl; 3 Institute of Electronic Materials Technology/Wolczynska str. 133, 01-919 Warsaw, Poland

**Keywords:** temperature sensors, flexible electronics, textronics, carbon nanotubes, fibers

## Abstract

The aim of this paper is to present research dedicated to the elaboration of novel, miniaturized flexible temperature sensors for textronic applications. Examined sensors were manufactured on a single yarn, which ensures their high flexibility and good compatibility with textiles. Stable and linear characteristics were obtained by special technological process and applied temperature profiles. As a thermo-sensitive materials the innovative polymer compositions filled with multiwalled carbon nanotubes were used. Elaborated material was adapted to printing and dip-coating techniques to produce NTC composites. Nanotube sensors were free from tensometric effect typical for other carbon-polymer sensor, and demonstrated TCR of 0.13%/K. Obtained temperature sensors, compatible with textile structure, can be applied in rapidly developing smart textiles and be used for health and protections purposes.

## 1. Flexible Sensors in Textile Applications

So called smart textiles are becoming more and more popular nowadays since their revolutionary concept significantly extends the potential field of applications. These products are frequently equipped with miniaturized, flexible electronic sensors, which improve greatly the functionality of integrated bio-parameter monitoring systems [1,2]. One of the most important groups of these devices are body temperature controlling systems. A correct, fast and accurate functioning of this system guarantees safety of the intelligent textile user, especially in protective wear [3]. Other sensors are also required for secure and effective emergency work, including chemical, movement and orientation detectors as well as breathing and cardiac monitors [4,5].

In this paper, the development of fully functional, flexible temperature sensors on separate yarns is presented. The elaborated devices are dedicated for direct incorporation into the fabric as the ultimate textronic integration step. Such sensors must meet some specific requirements. First of all they should be characterized by high static and dynamic accuracy in the human skin temperature range and provide easy integration into textile garments. This directly implies high flexibility and lightweight construction. Detection characteristics should be linear with possibly high temperature coefficiency. Encapsulation should be harmless to the human body, waterproof, and resistant to humidity and expected environmental challenges. It must be also resistant to chemicals used during dry cleaning of the textiles. Basing on these requirements flexible temperature sensors, operating in the 30–42 °C temperature range were designed and tested.

There are not many proposals for flexible temperature sensors, which could be easily integrated into textiles. Scientists from National Taiwan University have elaborated flexible temperature sensor arrays based on a graphite-polydimethylsiloxane composite [6]. These sensors were patterned on the flexible substrate and, depending on the content of graphite powder, different sensing temperature changes could be obtained. This solution can be used as a humanoid artificial skin for the sensation system of robots.

However, the most popular solution for temperature control in textronic systems is application of common digital sensors, for example DS18B20 integrated circuits [7]. The main advantages of the common integrated circuits application are linear characteristics, good accuracy and repeatability. It is one of the easiest temperature monitoring solutions, however the flexibility of these sensors is not assured. Therefore the proposed temperature sensors on fiber of higher flexibility can be serious competition for common sensors, especially for textronic applications.

## 2. Thermistor Structure

First the most suitable sensor substrate should be chosen. Examined fibers for analogue sensor cores could be divided into two groups: multifilaments and monofilaments. The first group was represented by basaltic and two types of carbon fibers. These materials are high temperature resistant, but their main disadvantage is insufficient coating adhesion. Copper, silver-plated and polyvinylidene fluoride (PVDF) fibers belong to the second group. They are characterized by sufficient temperature resistance, good flexibility and proper adhesion. The abovementioned fibers were examined precisely and their main properties are presented in Table 1. The temperature endurance measurements were performed at the range of 100–850 °C. However, such high temperature resistance is not necessary for curing polymer pastes, but in this case the active layer could be made of other components, for example metal oxides. Due to the plastic character of multifilament yarns, their diameters are given within allowable range.

**Figure 1.**
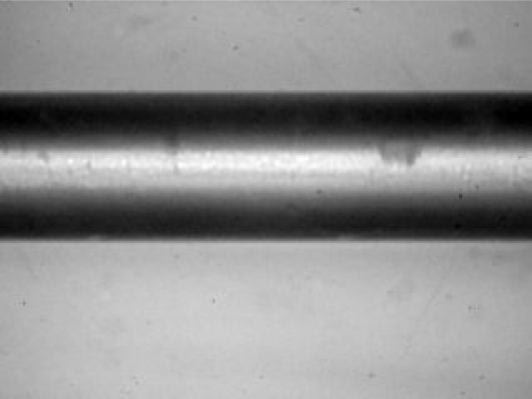
The PVDF monofilament fiber, magn. × 10.

The most appropriate fiber, the PVDF monofilament (Figure 1), was chosen as the sensor substrate owing to its higher flexibility and lower mass factor with sufficient thermal resistance. Its additional advantages are low cost, compatibility with weaving technology and additionally, dielectric and corrosive-proof character. Selected samples of fiber were of 2–6 cm length and 0.15 mm diameter.

**Figure 2.**
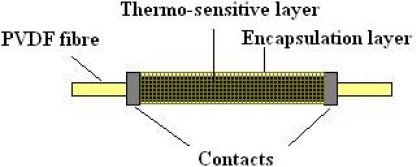
Thermistor structure.

Basing on the proposed construction, shown in Figure 2, the dielectric fibre was employed as the sensor base. The fibre was covered with a thermosensitive layer with ohmic contacts on both ends. At the end the thermistor was covered with an encapsulation layer. This option allowed omitting the additional technology step of sensor core isolating.

## 3. Materials: Thermo—Sensitive Pastes

Thermo-sensitive and polymer conductive pastes were placed and cured on the selected flexible fiber to form the active sensor area. The standard post—deposition thermal process of screen-printed pastes involves specific technology requirements, including sintering in 850 °C. Unfortunately polymer fibers are not resistant to such high temperatures, which are required for firing components of standard NTC or PTC pastes [8]. In the consequence of this lack of endurance thermo-sensitive paste should be applied as polymer composites. Thus the thermistor can only be produced through sintering metallic powder mixtures at temperatures lower than 200 °C. For this approach a new thermo-active material must be proposed and to meet this demand newly designed polymer based compositions were employed.

The first approach was based on graphite-polymer paste, which consists of rubber and polyethylene modified polystyrene as a binder material. These components were dissolved in organic solvents at elevated temperature, until a homogeneous consistence was obtained. The next step was the addition of carbon graphite filler and mixing in a three-roller mill. Rolling was performed until agglomerate sizes of below 10 μm were obtained [8]. Several series of sensors were produced and tested. These temperature sensors are characterized by high temperature coefficients, reaching 0.75%/°C in the 25–45 °C range. Unfortunately, sensors based on this kind of paste suffer from high tensometric effects. Sensor resistance varied significantly during stretching, bending and mechanical stresses, reaching 400% of initial value. This eliminated them from textronic applications. To get avoid this effect another paste composition was proposed.

The use of carbon-nanotubes improves the functionality of polymer composites by enhancing their strength and thermal or electrical conductivity [9,10]. Composites with carbon nanotubes can revolutionize structural materials design and production in construction elements [11,12]. Potential applications for electronic circuits fabricated with printing techniques are flexible electronics [13] and smart clothing [14] including functional elements (*i.e.*, printed transistors) [15,16] or biochemical sensors [17,18]. During previously conducted experiments related to carbon-nanotube layers [19] a strong resistance dependence on temperature was indicated, which allowed for experimenting in the textronic thermal sensory field.

**Figure 3.**
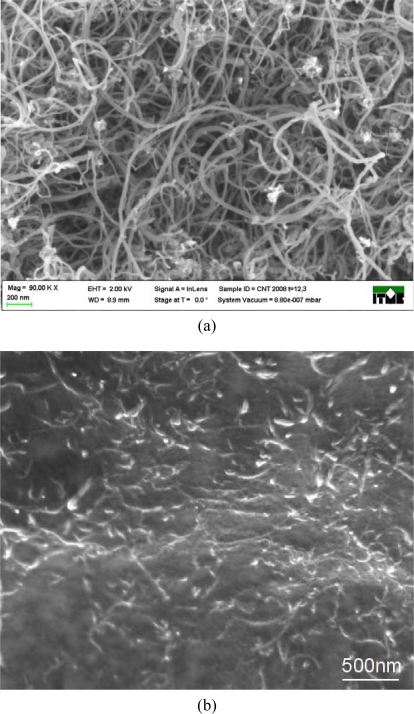
(a) HRSEM micrograph of MWCNT material used in screen-printing paste preparation. (b) HRSEM micrograph of MWCNT polymer layer.

In the reported investigation its authors elaborated a new polymer paste based on carbon nanotubes. Elaborated polymer compositions, containing carbon nanotubes, were prepared from commercially available materials. Multiwall carbon nanotube (MWCNT) material obtained by the CCVD method was used without any purification, or segregation. Diameter of nanotubes as well as their length were estimated from HRSEM observations (Figure 3). The mean diameter was estimated to be around 20–40 nm and length was in the 0.5–5 μm range although longer nanotubes were also observed. The material consisted mostly of carbon nanotubes, but other structures were observed, such as residues of metal catalyst (less than 4%) and amorphous carbon. One of the goals was to fulfill idea of mass production of low cost textronics devices. Therefore “as-prepared” material was used instead of segregated or purified CNTs, that can be several hundred times more expensive. Experiments with purified carbon nanotubes (above 99%) of similar characteristic dimensions resulted in comparable values of resistance of the polymer composites.

Multiwalled carbon nanotubes where added to vehicle consisting of poly(methylmethacrylate) (PMMA) polymer dissolved in an organic solvent. Polymer resin was a 12% butyl carbide acetate solution of M_W_ ∼350 k PMMA from Sigma-Aldrich. The dissolution process was conducted with a magnetic mixer for 48 h in 40 °C.

Carbon nanotubes were dispersed in the polymer resin to obtain final compositions. Since CNTs tend to agglomerate their dispersion in organic vehicle was the combination of several processes. The mixing process must be done very carefully since CNTs could be easily cut. CNTs were added to resin, premixed with pestle and mortar. The next step was ultrasonic stirring in a bath for 1 h to homogenize the mixture. The last procedure was milling on a three-roll-mill to break any remaining agglomerates. Components were mixed to obtain a homogeneous structure. Homogeneity was evaluated optically under an optical microscope, to verify if no agglomerates remained. Samples with macroscopic visible CNT agglomerates or areas with lack of CNTs were disqualified from further experiments. Depending on the carbon-nanotube concentration three types of compositions, were prepared. The weight content of CNTs in the polymer composition was 0.25%, 1% and 2% wt. nanotubes respectively. These solutions guarantee high initial resistance range and potentially various temperature coefficients. Properties of the used pastes are presented in Table 2.

**Figure 4.**
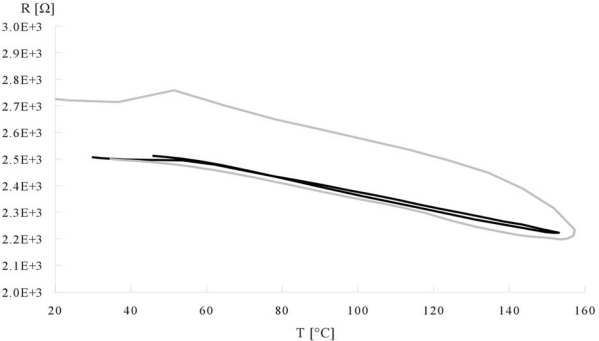
Resistance of stripes in function of temperature changes for 2% CNT samples after first (gray) and second (black) heating procedure.

Resistance *vs*. temperature measurements were taken in the range of temperatures from 20 °C to 160 °C in 20 second cycles. After this test samples were left to cool down to room temperature and tested again with the same procedure. Experimental results are presented in Figure 4.

While the global TC for polymer-nanotube samples was negative, local increase of resistance was observed around 50 °C. Also strong hysteresis was noticed for all CNT samples. This momentary increase of resistance and observed hysteresis are caused by local reorientation of polymer chains near the Tg (glass transition) temperature of the polymer base. This local effect in the polymer is causing a decrease of the probability of nanotube-nanotube connection, thus lowering the volume conductance in the layer and creating initial resistance growth. After a second heat up this effect is much smaller or even unnoticeable. This observation led us to suggest a method of TC stabilisation.

To obtain stable negative temperature coefficients a double thermal process was elaborated. Details of drying and annealing process of the deposited material are presented in Table 3. By the presented process proper temperature characteristics were obtained, however during the first test poor adhesion of the thermosensitive layers was obtained. Modification of the thermal process was adopted to eliminate this fault.

At this stage both phases, curing and annealing were performed subsequently without any time interval. This approach resulted in manufacturing of the sensor after a single temperature profile. As a result the sensitive material adhesion was significantly improved, while according to preliminary tests the thermal coefficient is preserved.

**Figure 5.**
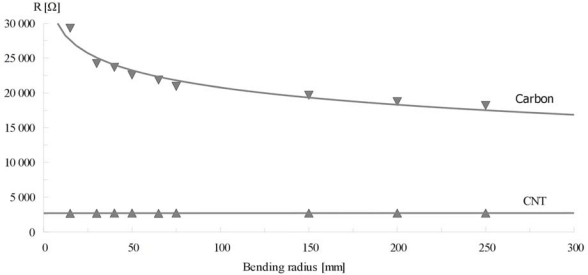
Resistance change of test stripes printed from nanotube-polymer and graphite-polymer compositions during bending cycle.

**Figure 6.**
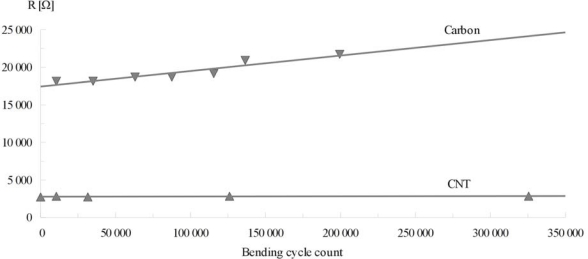
Resistance change of test stripes printed from nanotube-polymer and graphite-polymer compositions during cyclical bending.

Printed test stripes were also used for mechanical tests. Measurements of tensometric effect and resilience to cyclical bending, both factors crucial for textronic devices, were taken. Results of resistance measurements during bend radius change are presented in Figure 5 and results of cyclical bending tests are presented in Figure 6.

Strong tensometric effects are observed for stripes printed from graphite-polymer compositions. Stripes made from CNT compositions remained stable during the whole test. This effect is related to the shape of the functional phase particles. While graphite grains were losing physical contact because of small number of connections between separate grains that can be approximated by spheres, in the case of nanotubes, which are structures with length-to-diameter aspect in range of 100 to 1,000, the probability of contact loss is very small, thus resistance of obtained conductive composite remained unchanged.

As in the previous case test stripes made from CNT compositions remained stable during whole cyclical bending test. Again nanotube-polymer composites are resilient to this type of damage. Graphite-polymer composites also maintained resistance change at a fair level, but after 200,000 cycles more than 10% growth of resistance is noted.

## 4. Manufacturing of the Devices and Encapsulation Process

**Figure 7.**
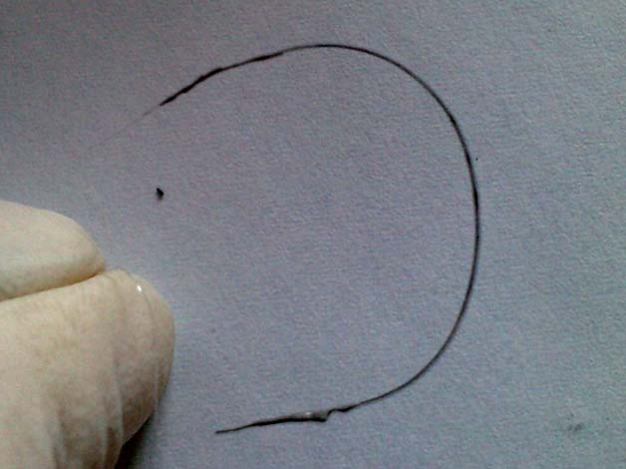
Flexible temperature sensor on yarn, contacts made of polymer conductive paste.

Manufacture of effective electric contacts for nanotube layers on polymer yarns is a challenging task since the classical soldering technology cannot be employed for this application. For the first approach polymer conductive paste with silver metallic filler ITME L-121, was chosen as the contact material. It allows obtaining low resistances in the range of 10^−2^–10^−1^ Ω with preserved high flexibility at an acceptably low process temperature (curing at 120 °C). While the resistance of silver contacts is five orders of magnitude lower than for polymer-CNT thermistor layer, the plausible tensometric effect in L-121 contact can be negligible. The structure of an elastic temperature sensor on a single yarn, with conductive polymer contacts is shown in Figure 7.

**Figure 8.**
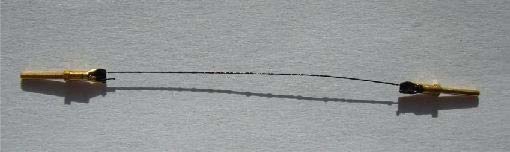
Flexible temperature sensor on yarn with contact connectors.

The paste showed better compatibility with CNT polymer paste. Polymer conductive contacts appeared sufficient for the first sensor measurements, however their high thickness, relatively low adhesion and difficulties during connection with other conductive materials suggested the need for further contact development [20]. Therefore a new contact approach was proposed. In the end the authors decided to evaporate selective metallic contacts. For laboratory measurements metal clamps were attached. The finished thermistor is presented in Figure 8.

The obtained structure is sensitive to environmental dangers therefore for proper functioning in textiles it demands appropriate encapsulation. To fulfil this demand silicon paste was chosen as an isolating material. This solution appeared partially efficient, however the high silicon density resulted in a thick paste layer (circa 300 μm). This decreased the yarn flexibility rapidly and made textile fabrication impossible. Thus other approach, based on plastic isolating varnish was employed. Isolating plastic varnish by CRC was sprayed on the surface of the sensor at the active area, excluding contact pads. This way elastic, a transparent and dielectric layer of 200 nm was obtained. The electrical isolation quality was confirmed by resistance measurements.

During the exploitation of temperature sensors on yarns, encapsulated by dielectric polymer coatings and deposited by spray techniques, some failures were detected. It appeared that solvents based on acrylic acids, present in the spray presnet a serious danger for nanotube layers. Therefore, an alternative technique of encapsulation appeared necessary. The final production series of sensors was achieved by rinsing the yarn with a liquid silicon paste, producing an acceptably thin surface coating, which resulted in a preserved nanotube layer structure.

## 5. Thermistor Parameters

**Figure 9.**
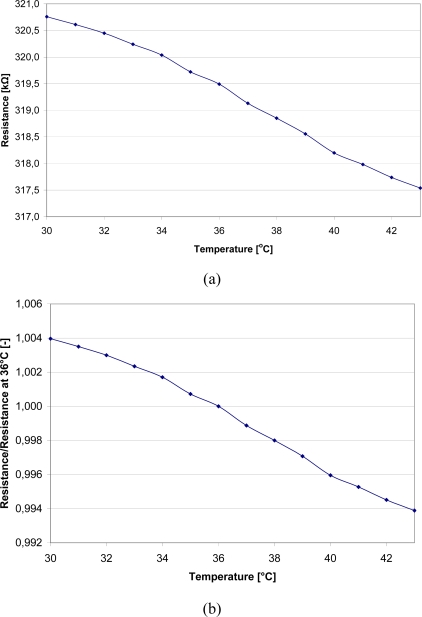
Real (a) and normalized (b) temperature—resistance characteristics of performed polymer elastic temperature sensors, manufactured on single yarn.

The initial thermistor resistance can be easily adjusted for further conversion by sensor length, carbon-nanotube content and also by matching the proper temperature profile. Figure 9 presents real and normalized characteristics of performed sensor. The active material was carbon-nanotube paste with 2% carbon-nanotube content.

The above results were obtained for 2% CNT material sensor under calorimetric conditions. All measurements indicated the expected resistance change with temperature however, some non-linearity in the characteristic flow suggests the need for thermal process modifications. Further manufacturing and measurement experiments are planned.

## 6. Conclusions

**Figure 10.**
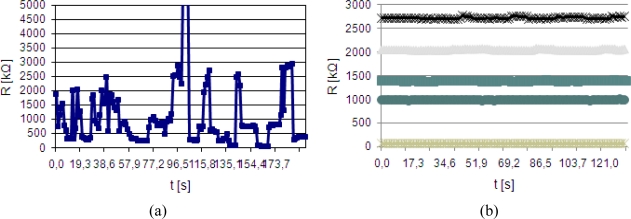
The influence of dynamic mechanical stress on temperature sensors resistivity made of graphite-polymer paste (a) and CNT paste (b).

Based on the presented technology a series of miniaturized temperature sensors on single yarns were manufactured. These sensors could be easily integrated into textronic inner garments due to their small dimensions and good flexibility. The measurement range of the obtained sensors allows for human skin temperature control in an extended temperature range (30 °C–45 °C) with proper linearity and temperature coefficients, dependent on the composition of the thermo-sensitive paste used. The first carbon-polymer paste was characterized by a high PTC temperature coefficient, equal to 0.75%/K, whilst the second paste based on carbon-nanotube (CNT) components was equal to 0.13%/K. The earlier demonstrated tensometric effect of carbon-polymer sensors disqualifies their application in flexible electronics. As the elimination of this parasitic effect carbon-nanotube material was used and successfully tested as an active layer (Figure 10).

This novel solution, verified by practical experiments, guarantees stable control of human skin temperature and the possibility of wide sensor applications in textronics.

## Figures and Tables

**Table 1.** Properties of examined fibers.

	**Structure**	**Diameter [mm]**	**Temperature resistance [°C]**
Carbon fiber—1st type	Multifilament	0.07–0.13	850
Carbon fiber—2nd type	Multifilament	0.08–0.14	850
Basaltic fiber	Multifilament	0.04–0.10	550
Copper fiber	Monofilament	0.05	350
Silver-plated fiber	Monofilament	0.05	300
Polyvinylidene fluoride fiber	Monofilament	0.15	185

**Table 2.** Electrical and thermal properties of elaborated thick film stripes.

**Composition**	**Curing temperature [°C]**	**Curing time [h]**	**Stripe resistance [kΩ]**	**TC [ppm/K]**
Carbon polymer	120	0.3	17.4	7,500
CNTs 0.25%	120	1	35.2	1,010
CNTs 1%	120	1	6.52	1,780
CNTs 2%	120	1	2.73	2,110

**Table 3.** Thermal treatment of nanotube paste.

**Process No.**	**Process type**	**Process temperature**	**Duration**	**Obtained Thermal Coefficient**
1	Curing	120 °C	1 h	Positive, instable
2	Annealing	120 °C	1 h	Negative, stable
